# Supplementary material for: The Influence of Adherence to Orthosis and Physiotherapy Protocol on Functional Outcome after Proximal Humeral Fracture in the Elderly
Source: J Clin Med. 2023 Feb 22;12(5):1762. doi: 10.3390/jcm12051762 (PMC10003098; doi:10.3390/jcm12051762)
Supplement: Supplementary file 1 [file jcm-12-01762-s001.zip › physiotherapy protocols and the questionnaire/PATIENT QUESTIONNAIRE.pdf]

LMU KLINIKUM
Muskuloskelettales Universitätszentrum
München - MUM

MUM

PATIENT QUESTIONNAIRE

„Influence of compliance in geriatric trauma patients with proximal humerus fracture“

First letter given name: _________ first letter family name: _________ birth date: __.__.19__

Study subject number: __________ Date of trauma: _________

FU: 6 weeks ☐ 3 months ☐ 6 months ☐ 12 months ☐

To be completed for the 6-week follow-up:

Orthosis worn as recommended in the protocol: yes ☐ no ☐

If "no" give reason (multiple choice possible):

Removal of orthosis after _____ days after trauma/if not determinable after _____ weeks

☐ Painlessness

☐ Increased pain during immobilization

☐ Unrecognizable benefit

☐ difficulties with the application of the orthosis

☐ discomfort while wearing the orthosis

☐ soft tissue irritations

☐ other discomfort/intolerances

☐ hygiene problems

☐ switch to a different orthosis (________________)

Partial application of the orthosis? ☐ yes ☐ no

If yes how many hours per day? ________

If yes how many hours per night? ________

How many physiotherapy sessions did the patient have up to date? ________

If no physiotherapy or less than 6 sessions were done, why? (multiple choice possible)

- ☐ no free appointments
- ☐ unrecognizable benefit
- ☐ due to immobility no possibility to get to appointments /no home visits
- ☐ cognitive deficits

---

functional outcome (to be filled in at every follow-up visit)

pain (VAS 1-10) ________

Constant score: ________ points

https://www.orthopaedicscore.com/scorepages/constant_shoulder_score.html

---

radiological outcome (to be filled in at every follow-up visit)

CCD-angle on the a.p. X-ray [°]: ________ °

Complications and revision surgery since last follow-up visit:

____

____

____

Comments:

____

____

____

____

____

_________________________ _________________________________

Date of follow-up visit Name of physician
