# Supplementary material for: The Influence of Adherence to Orthosis and Physiotherapy Protocol on Functional Outcome after Proximal Humeral Fracture in the Elderly
Source: J Clin Med. 2023 Feb 22;12(5):1762. doi: 10.3390/jcm12051762 (PMC10003098; doi:10.3390/jcm12051762)
Supplement: Supplementary file 1 [file jcm-12-01762-s001.zip › physiotherapy protocols and the questionnaire/Physiotherapy protocol PHF conservative.pdf]

MUM | Muskuloskelettales Universitätszentrum München

Proximal humerus fracture

Conservative treatment

Patient: ______________________________ **date of trauma:** ____________

time	limitation to ROM	Physiotherapy
1.– 5.week	Flexion/Ext. 60-15-0<br>Abduktion/ADD. 60°-15-0;<br>ER/IR 20-0-20	Simple arm sling<br>Scapulamobilisation and<br>–stabilisation passive/active<br>Assisted/active ROM excercises; manual mobilisation and stretching of parascapular Mm.;<br>Up to 3. week gaining MTW 3/5<br>Manual lymphatic drainage
3.- 6. week	Training of everyday functions below 90° flex. And abd. with bodyweight (MTW 3/5);<br>Rotation from 5. week on without limitation	
from 7. week	No limitations	Strengthening of the delta- and Scapulamuscles >3/5,<br>increase resistance
from 12. week	Free motion, full load	

X-ray controls after 1, 3 and 6 weeks in the outpatient clinic:

Großhadern:	Innenstadt:
PD Dr. T. Helfen; PD Dr. F. Gilbert	PD Dr. T. Helfen; PD Dr. F. Gilbert
Klinikum Großhadern Chirurgische Poliklinik B	LMU Klinikum Innenstadt
Marchioninistr. 15 81377 München	Ziemssenstr. 5 80336 München
wednesday 9.00 a.m. - 1.00 p.m.;	monday 9.00 a.m. - 3.00 p.m.;
appointments: phone: +49-89-4400-73505; Fax -76505	appointments: phone: +49-89-4400-54040; Fax-52745
Termin-MUM@med.uni-muenchen.de	Termin-MUM@med.uni-muenchen.de
www.MUM-LMU.de	www.MUM-LMU.de
