# Supplementary material for: The Influence of Adherence to Orthosis and Physiotherapy Protocol on Functional Outcome after Proximal Humeral Fracture in the Elderly
Source: J Clin Med. 2023 Feb 22;12(5):1762. doi: 10.3390/jcm12051762 (PMC10003098; doi:10.3390/jcm12051762)
Supplement: Supplementary file 1 [file jcm-12-01762-s001.zip › physiotherapy protocols and the questionnaire/Physiotherapy protocol PHF surgical.pdf]

MUM | Muskuloskelettales Universitätszentrum München

Proximal humerus fracture
surgical treatment

Patient: ______________________________ date of surgery: __________

time	limitation to ROM	Physiotherapy
1.– 2.week	Assisted/active ROM excercises up to<br>Flexion/Ext. 60°<br>Abduktion/Add. 60°<br>ER/IR 20-0-20°	Simple arm sling for analgesia<br>Scapulamobilisation and<br>–stabilisation passive/active<br>manual mobilisation and stretching of parascapular Mm.;<br>Manual lymphatic drainage
3.- 4. week	Flexion/Abduction 80° (assisted actively)<br>Extension/Add. (active)<br>ER/IR 30-0-30° (assisted actively)	see above<br>additional scar treatment when stitches removed
5.- 6. week	Flexion/Abduction 120° (assisted active)<br>Extension/Add. without limitation (active)<br>ER/IR without limitation (isometric active)	Additionally swimming pool possible
from 7. week	Pain adapted strengthening<br>No limitations	Strengthening of the delta- and Scapulamuscles >3/5,<br>coordination- and stabilisation training

X-ray controls after 6 weeks in the outpatient clinic:

Großhadern:<br>PD Dr. T. Helfen; PD Dr. F. Gilbert<br>Klinikum Großhadern Chirurgische Poliklinik B<br>Marchioninistr. 15 81377 München<br>wednesday 9.00 a.m. - 1.00 p.m.;<br>appointments: phone: +49-89-4400-73505; Fax -76505<br>Termin-MUM@med.uni-muenchen.de<br>www.MUM-LMU.de	Innenstadt:<br>PD Dr. T. Helfen; PD Dr. F. Gilbert<br>LMU Klinikum Innenstadt<br>Ziemssenstr. 5 80336 München<br>monday 9.00 a.m. - 3.00 p.m.;<br>appointments: phone: +49-89-4400-54040; Fax-52745<br>Termin-MUM@med.uni-muenchen.de<br>www.MUM-LMU.de
