# Supplementary material for: The Influence of Adherence to Orthosis and Physiotherapy Protocol on Functional Outcome after Proximal Humeral Fracture in the Elderly
Source: J Clin Med. 2023 Feb 22;12(5):1762. doi: 10.3390/jcm12051762 (PMC10003098; doi:10.3390/jcm12051762)
Supplement: Supplementary file 1 [file jcm-12-01762-s001.zip › physiotherapy protocols and the questionnaire/Physiotherapy protocol PHF tuberosity.pdf]

Proximal humerus fracture with greater tuberosity

Conservative & surgical

Patient: ____ date of surgery/trauma: ____________

time	limitation to ROM	Physiotherapy
1.– 4.week	Assisted/active ROM excercises up to<br>Flexion/Ext. 60-15-0°<br>Abduktion/Add. 60-15-0°<br>ER/IR 20-0-20°	Abduction orthosis<br>Scapulamobilisation and<br>–stabilisation passive/active<br>manual mobilisation and stretching of parascapular Mm.;<br>Manual lymphatic drainage
5. week	Flexion/Abduction 80° (assisted actively)<br>Extension/Add. Up to 0° (active)<br>ER/IR 30-0-30° (assisted actively)	see above<br>additional scar treatment when stitches removed<br>abduction orthosis durigng night
6. week	Flexion/Abduction 120° (assisted active)<br>Extension/Add. without limitation (active)<br>ER/IR without limitation (isometric active)	Additionally swimming pool possible<br>Strengthening of the delta- and Scapulamuscles >3/5,<br>coordination- and stabilisation training

X-ray controls after 6 weeks in the outpatient clinic:

Großhadern:	Innenstadt:
PD Dr. T. Helfen; PD Dr. F. Gilbert	PD Dr. T. Helfen; PD Dr. F. Gilbert
Klinikum Großhadern Chirurgische Poliklinik B	LMU Klinikum Innenstadt
Marchioninistr. 15 81377 München	Ziemssenstr. 5 80336 München
wednesday 9.00 a.m. - 1.00 p.m.;	monday 9.00 a.m. - 3.00 p.m.;
appointments: phone: +49-89-4400-73505; Fax -76505	appointments: phone: +49-89-4400-54040; Fax-52745
Termin-MUM@med.uni-muenchen.de	Termin-MUM@med.uni-muenchen.de
www.MUM-LMU.de	www.MUM-LMU.de
